# W-Band Through-Wall Radar Using a High-Gain Frequency-Scanning SSPP Antenna

**DOI:** 10.3390/mi16111276

**Published:** 2025-11-13

**Authors:** Zhenfeng Tian, Jinling Zhang, Wang Yan, Yingzhe Wang, Xiongzhi Zhu, Xiaoqing Zhang, Pan Pan

**Affiliations:** 1 College of Electronic Engineering, Beijing University of Posts and Telecommunications, Beijing 100876, China; tianzf@bupt.edu.cn (Z.T.); wangyanian@bupt.edu.cn (W.Y.); wangyingzhe@bupt.edu.cn (Y.W.); 2 College of Electrical and Information Engineering, Zhengzhou University, Zhengzhou 450001, China; zhuxiongzhi@zzu.edu.cn; 3 The National Key Laboratory of Science and Technology on Vacuum Electronics (NKLST-VE), Beijing Vacuum Electronics Research Institute (BVERI), Beijing 100015, China; zhangxiaoqingbveri@163.com (X.Z.); p-pan@hotmail.com (P.P.)

**Keywords:** high-gain beam-scanning antenna, W-band through-wall radar (TWR), sinusoidally modulated reactance surfaces (SMRS), through-wall imaging

## Abstract

This letter presents a high-gain frequency-controlled beam-scanning antenna specifically designed for through-wall radar (TWR) applications in the W band. The antenna leverages the leaky-wave radiation generated by spoof surface plasmon polaritons (SSPPs) propagating on sinusoidally modulated reactance surfaces (SMRS). Periodically arranged quasi-H-shaped metallic cells are employed to achieve beam scanning. The integration of a flared structure at the apex of the designed SSPP antenna results in a significant gain enhancement, yielding an approximate increase of 10 dB. From 92.8 to 97.6 GHz, the antenna exhibits a reflection coefficient of |S11| < −10 dB, provides a high scanning rate of 4.05°/%, and achieves a realized gain of 20.9 dBi. This design eliminates the necessity for mechanical rotators and phase shifters that are typical in traditional TWR systems, significantly reducing system complexity and cost. A vehicle-mounted W-band TWR system was developed, integrating the designed SSPP antenna and employing linear frequency modulation technology to emit millimeter-wave signals for electronic scanning detection. With an economical and efficient design approach, testing has demonstrated that the system can perform through-wall imaging at a distance of 10 m, both in stationary and in motion conditions.

## 1. Introduction

TWR systems have attracted considerable attention in recent years due to their potential applications in military counter-terrorism operations, urban security inspections, and emergency search-and-rescue missions [1,2,3,4]. These systems employ electromagnetic waves to penetrate obstacles such as walls, thereby facilitating the detection of targets or areas of interest located behind these barriers. Presently, most TWR systems function within frequency bands below 10 GHz, primarily due to power limitations and wall losses. The constraints associated with low-frequency bandwidth render the achievement of high range resolution particularly challenging [5,6,7,8].

To achieve high resolution of TWR systems, the introduction of the W-band TWR has revitalized traditional design approaches [9]. However, realizing this high-frequency TWR system necessitates high-power synthesis to offset the losses, requiring antennas to exhibit high-power capacity. The metal waveguide structure presents a competitive solution. Radar system detection typically utilizes either mechanical scanning or phased array methods [10,11,12]. Although mechanical scanning offers a straightforward design, it suffers from slower scanning speeds and vulnerability to wear in moving parts, which can compromise stability and reliability over extended use. In contrast, while phased array antennas enable rapid beam scanning, they encounter significant design challenges—such as the complexities of phase shifters at W-band frequencies and increased overall complexity. The feasibility of utilizing frequency scanning antennas for radar imaging is demonstrated in [13]. A low-loss W-band frequency scanning antenna based on waveguide slot structures is presented. Similarly, by optimizing the arrangement and dimensions of the slots or using the design of a continuous transverse slot array antenna in the metal waveguide, the frequency scanning antenna can also be achieved [14,15]. However, the scanning angle range of the conventional studies relies on a very large bandwidth, resulting in low scanning rates, necessitating other RF components to achieve a very wide operational bandwidth, which poses significant challenges in the practical design of W-band radar transceivers.

SSPPs are surface-bound electromagnetic waves that propagate in the microwave and terahertz frequency range. Their electric fields can be strongly confined at the metal-dielectric interface, exhibiting significant dispersion characteristics [16]. A common approach to achieving beam scanning antennas using SSPPs is to load parasitic elements that excite radiation fields through coupling effects with the main transmission line, but this method suffers from high transmission loss and low radiation efficiency [17,18,19,20]. Another approach is to periodically modulate the geometric features of the SSPPs transmission line itself, which can enable radiation without the need for additional loading structures. This method is more compact and allows for flexible modulation, making beam scanning easily achievable [21,22,23]. However, the aforementioned studies primarily focus on lower frequency ranges, often using printed circuit board (PCB)-based implementations, leading to significant dielectric losses, with a risk of dielectric breakdown and surface discharge phenomena under high-power operating conditions, thus limiting power handling and low gain.

**Figure 1:**
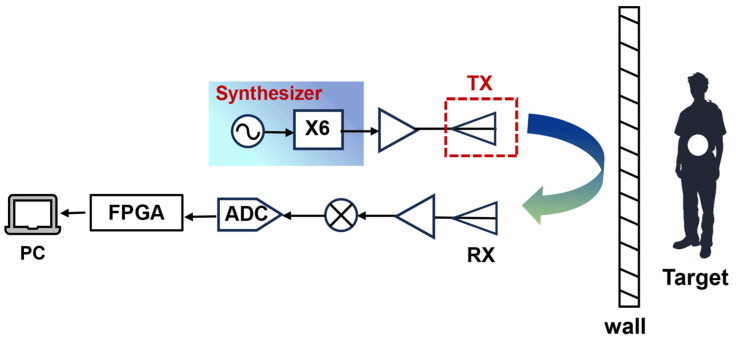
Schematic block diagram of TWR.

Figure 1 illustrates a simplified schematic of the TWR system. W-band signals are emitted by the transmitting antenna after undergoing frequency synthesis and amplification, while the receiving chain collects and processes the echo signals. This study introduces a leaky wave antenna based on SSPPs for use as the transmitting antenna in TWR applications. A high-gain frequency-controlled beam-scanning antenna loaded with a flared radiation structure is designed. The antenna features a fully metallized structure that provides significant advantages in terms of high-power capacity. It operates without the necessity for additional multi-channel feeding networks or phase shifters. This design achieves a scanning rate of 4.05°/% across a bandwidth of 92.8 to 97.6 GHz, with a gain of up to 20.9 dBi. Consequently, this antenna effectively reduces both the complexity and cost of W-band TWR, offering an efficient and reliable solution for regional scanning detection. This study presents the implementation of a W-band TWR system based on the linear frequency modulation technique. The system has validated the feasibility of frequency control and beam scanning detection functionalities utilizing the SSPP antenna. Furthermore, the radar’s perception performance was evaluated in both stationary and vehicle-mounted configurations, thus confirming the prototype’s effectiveness under diverse conditions.

## 2. Materials and Methods

### 2.1. Configuration

**Figure 2:**
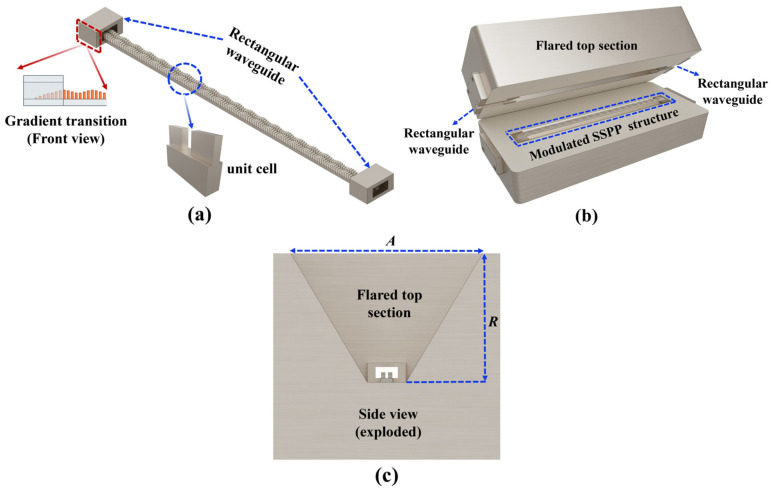
(**a**) Schematic diagram of the fully metallic SSPP antenna. (**b**) SSPP antenna with flared section. (**c**) Side view of the flared section, where *A* = 22 mm and *R* = 15 mm.

Figure 2 illustrates the two SSPP beam-scanning antennas discussed in this paper. Figure 2a presents a fully metallized frequency-scanning antenna composed of sinusoidally modulated SSPP units. The antenna’s feeding port uses a standard WR-10 waveguide flange. The metal gradient transition design facilitates the efficient transition from the TE mode of traditional rectangular waveguide to the TM mode of SSPP transmission. The high-gain SSPP antenna depicted in Figure 2b comprises two main components: the sinusoidally modulated SSPP antenna and the flared structure at the top. The flared top region is free space (air), and the surrounding body is machined aluminum with gold plating. The antenna’s terminus is attached to a matching load made of silicon carbide, which exhibits high thermal conductivity and stability, and is intended to absorb residual energy, mitigate the effects of reflected waves, and optimize impedance matching. The overall dimensions of the antenna are 58 mm × 26 mm × 24 mm.

### 2.2. Theoretical Analysis

**Figure 3:**
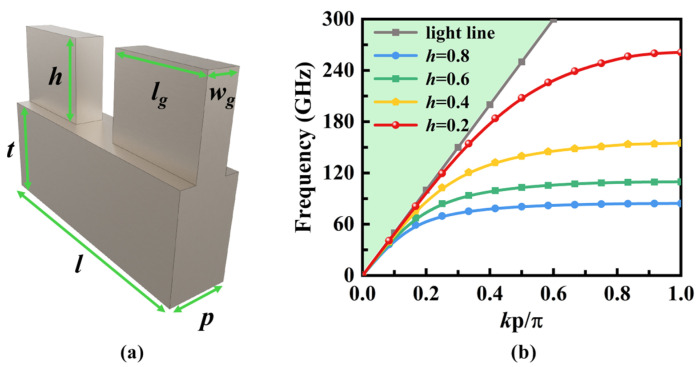
(**a**) Configuration of the quasi-H-shaped SSPP unit, where *p* = 0.4 mm, *l* = 1.5 mm, *l_g_* = 0.6 mm, *w_g_* = 0.2 mm, *t* = 1 mm. (**b**) Dispersion curves of the SSPP unit with different *h* (*h* in mm).

The SSPP unit design is illustrated in Figure 3a. The structure comprises dual symmetric metallic protrusions of identical geometric dimensions, forming a quasi-H-shaped SSPP unit. The cutoff frequency can be adjusted by controlling the height *h* as shown in Figure 3b. It is apparent that as the height h increases, the cutoff frequency decreases. For a given frequency, an increase in *h* results in a larger wavenumber along the propagation direction.

**Figure 4:**
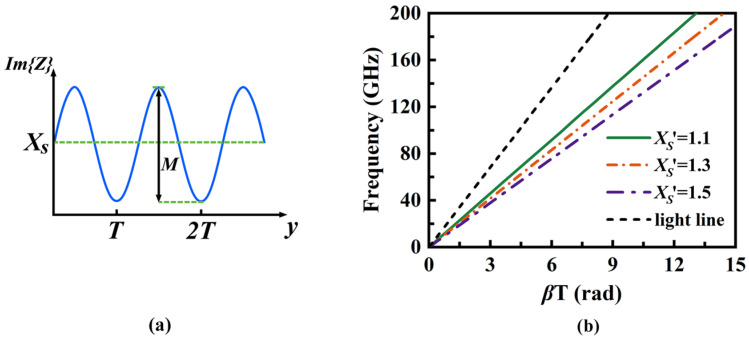
(**a**) Sinusoidal form of surface reactance. (**b**) Dispersion curves of the SMRS with different *Xs*′.

To guide energy from the transmission mode to the radiation mode, a periodic modulation design method based on the theory of SMRS is employed. SMRS stands for sinusoidally modulated reactance surface (SMRS). An SMRS is a surface whose reactance is varied sinusoidally along a given coordinate, enabling coupling between spatial harmonics and surface-wave modes [24]. This modulation periodically perturbs the propagation environment of SSPP, converting bound slow-wave modes into leaky fast-wave modes. As shown in Figure 4a, assuming the electromagnetic wave propagates along the *y*-axis, the distribution of surface impedance *Z* in the propagation direction can be described as follows:

(1) $$Z(y)=jX_{s}\left[1+M\cos(\frac{2\pi y}{T})\right]$$

The surface profile fluctuates around the average surface reactance *Xs* with a modulation factor *M* and period a *T*. As illustrated in Figure 4b, when *T* = 2.8 mm and *M* = 0.3, *β* increases alongside the normalized average surface reactance *Xs*′. If the modulation factor is sufficiently small, the wavenumber *k* (Bloch wavenumber) can be approximated [25].

(2) $$k\approx k_{0}\sqrt{1+{X_{s}}^{\prime 2}}\approx \beta$$

### 2.3. Realization of SSPP LWA

**Figure 5:**
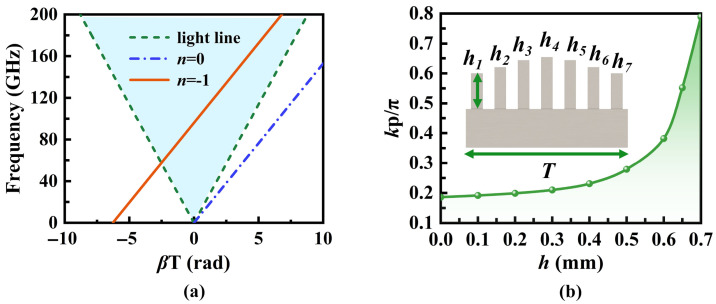
(**a**) Brillouin diagram of the *n* = 0 and *n* = −1 modes for an SMRS with *Xs*′ = 1.1, *M* = 0.3, and *T* = 2.8 mm. (**b**) Phase shift in the SSPP unit with varying heights.

The combined Floquet and SMRS theories suggest that SSPP surface waves can be expanded in space as a series of *n* harmonics. In the fast wave region, radiation occurs as depicted in Figure 5a, with the *n* = −1 mode being the primary contributor, which can be obtained by shifting the curve for *n* = 0. Based on the periodic variations in surface impedance, the modulated heights of the SSPP unit are determined as shown in Figure 5b and Table 1. The spatial harmonics are expressed as:

(3) $$\beta_{N}=\beta_{0}\pm \frac{2n\pi }{T}$$

**Figure 6:**
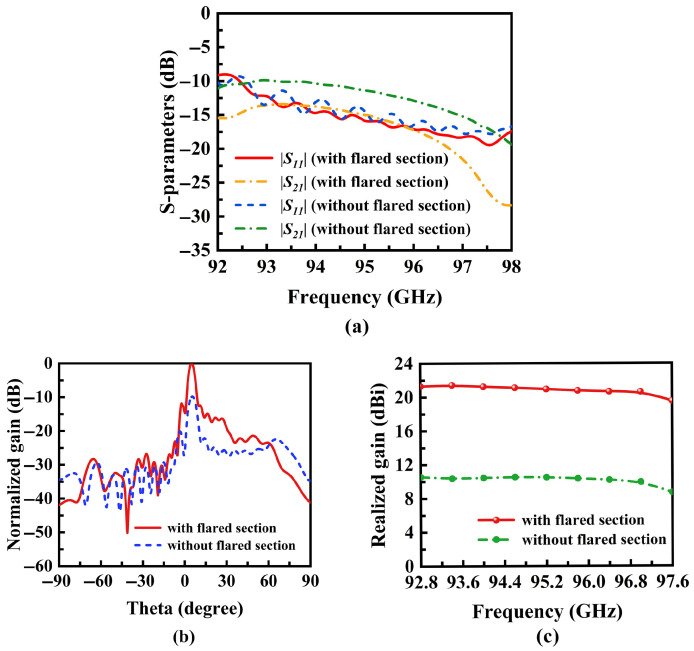
(**a**) Simulated S-parameters of SSPP antennas. (**b**) Simulated normalized radiation patterns at a frequency of 92.8 GHz. (**c**) Simulated gain curve of the proposed antenna.

We conducted a comprehensive design of the antennas using full-wave simulations in Ansys HFSS. The simulation results for antennas with and without the top structure were compared, as illustrated in Figure 6. Figure 6a presents similar results with |S11| and |S21| consistently below −10 dB. The antenna with the flared section concentrates radiation energy more effectively, improving directionality as shown in Figure 6b,c. The normalized radiation patterns show an approximately 10 dB increase in antenna gain. When the frequency is fixed, both antennas exhibit identical beam steering angles. This approach enables high-gain beam scanning, with the gain level adjustable by modifying the size and height of the tapered aperture to meet specific engineering requirements.

## 3. Results

### 3.1. Experimental Validation of the Proposed Antenna

**Figure 7:**
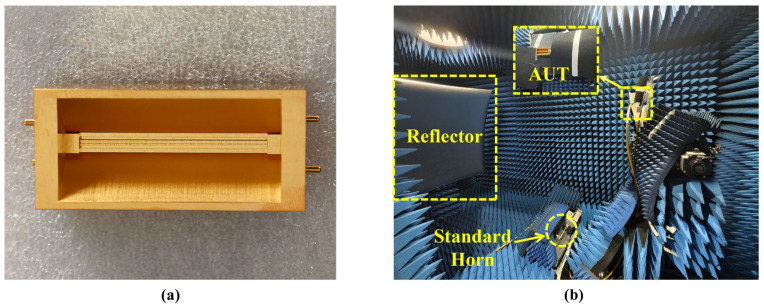
(**a**) Fabricated prototype of the proposed antenna. (**b**) Far-field measuring environment.

The prototype of the high-gain frequency-scanning antenna designed, along with the test scenario, is depicted in Figure 7. The antenna developed in this study is constructed from a high thermal conductivity, low-density aluminum alloy, which is shaped through computer numerical control (CNC) machining. The production tolerance was set at 5 µm, ensuring that the dimensional deviation from the specified values remains within this threshold, which is considered acceptable for structural applications [14,26,27]. To improve surface conductivity and oxidation resistance, the surface undergoes an electroplating treatment, wherein a gold layer approximately 1 μm thick is deposited to achieve superior electrical performance and environmental adaptability.

**Figure 8:**
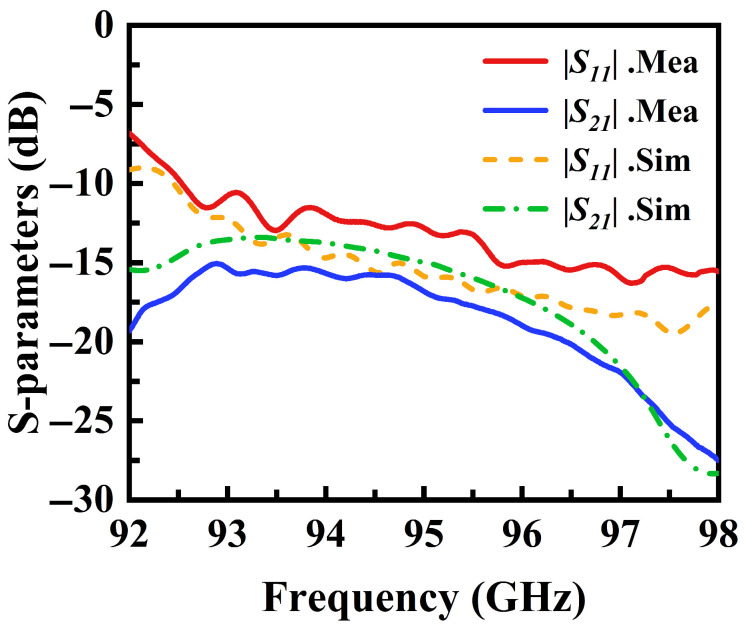
Simulated and measured results for S-parameters.

Figure 8 presents the simulated and measured S-parameter curves of the designed antenna. The close agreement between the curves demonstrates the consistency between simulation and measurement. Impedance matching is well-maintained across the entire operational frequency band, with the reflection coefficient |S11| consistently below −10 dB and the transmission coefficient |S21| below −13 dB. The |S21| denotes the guided transmission from the antenna feed port to the antenna termination. The minor discrepancies between the measured and simulated curves can be attributed to variations in the manufacturing process and factors such as the test waveguide connectors, which are considered reasonable and acceptable.

**Figure 9:**
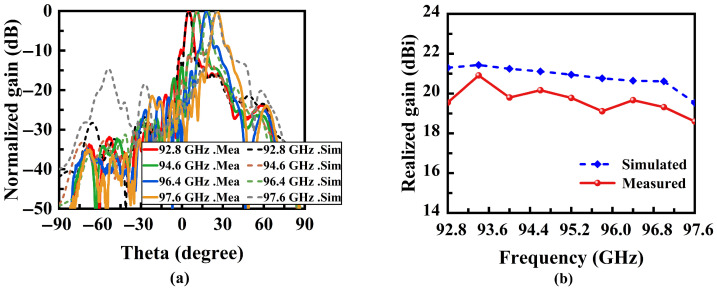
(**a**) Simulated and measured normalized radiation patterns. (**b**) Simulated and measured results for realized gain.

Figure 9a shows the normalized far-field radiation patterns at different frequencies, highlighting the variation in the antenna’s beam angle under different frequency conditions. At frequencies of 92.8 GHz, 94.6 GHz, 96.4 GHz, and 97.6 GHz, the beam directions are controlled at 5.2°, 11°, 19°, and 25.6°, respectively, closely matching theoretical predictions and yielding a scanning rate of 4.05°/% within the transmission frequency band. The sidelobe level remains consistently below −10 dB. The proposed antenna achieves a peak gain of 20.9 dBi. The measured gain is approximately 1.1 dB lower than the simulated value on average, as depicted in Figure 9b. This discrepancy arises from practical loss mechanisms not captured in ideal simulations, chiefly: assembly and alignment errors (including flange and gap losses), increased conductor loss due to surface roughness and finite conductivity at millimeter wave frequencies, and measurement uncertainties such as reference-antenna accuracy, connector and adaptor insertion loss and mismatch. Together, these effects account for the observed reduction in measured gain [27,28].

Table 2 provides a comparative overview of the research findings on relevant antennas as reported in the existing literature. Although references [13,14] employ metallic waveguide structures with high-power capacity, their frequency scanning range heavily depends on a large bandwidth, resulting in a low scanning rate within a certain bandwidth. Consequently, the beam scanning range is limited within the narrow operating bandwidth of TWR. While the antennas identified in references [22,25] demonstrate advantages in terms of flattening, they are unsuitable for high-power scenarios and also suffer from a low scanning rate. In contrast, the SSPP antenna proposed in this paper features a fully metallic design that enhances its power capacity and enables operation within the W band, exhibiting significant high-gain advantages.

### 3.2. Design and Implementation of the TWR System

**Figure 10:**
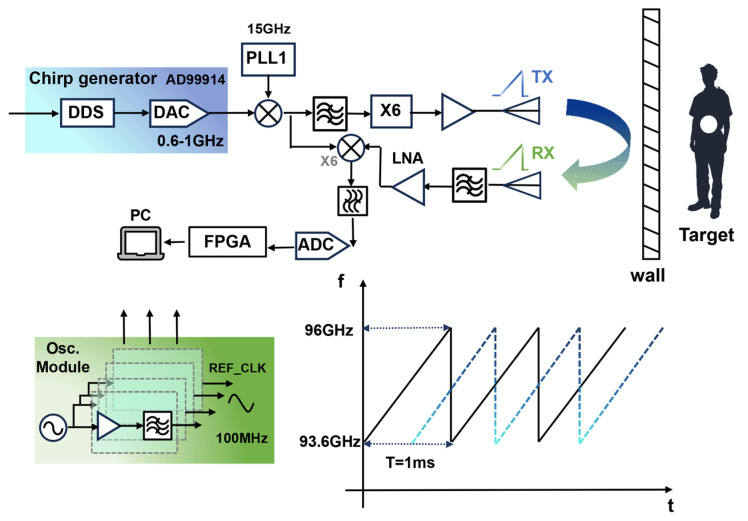
System architecture of the proposed W-band TWR transceiver.

The designed high-gain frequency scanning antenna is employed as the transmitting antenna for the radar system. The through-wall radar system transmits a linear frequency modulation signal, which aligns effectively with the beam control method of the antenna. This alignment facilitates frequency-controlled scanning detection using the antenna. To validate the frequency-controlled beam-scanning antenna in a practical through-wall radar, the overall design of the through-wall radar system integrated with this antenna is described. The W-band through-wall radar architecture is shown in Figure 10. The frequency source uses direct digital synthesis (DDS): an AD9914 generates a 600–1000 MHz linear frequency-modulated (LFM) chirp with a 1 ms period. The chirp is mixed with a 15 GHz signal from a phase-locked loop (LMX2594, Texas Instruments, Dallas, TX, USA). After mixing, a bandpass filter suppresses the local-oscillator and down-converted signals, preserving the up-converted signal at 15.6–16 GHz. This signal is then multiplied by six to reach the W-band, amplified in a final stage, and radiated through the designed high-gain SSPP antenna for scanning. Echoes from targets behind the wall are received by a standard horn antenna; following filtering, low-noise amplification, and mixing, they are converted to baseband using a second local oscillator (LO) from the transmit chain. Because the LO and echoes share the same chirp slope, this stage simultaneously achieves dechirping.

**Figure 11:**
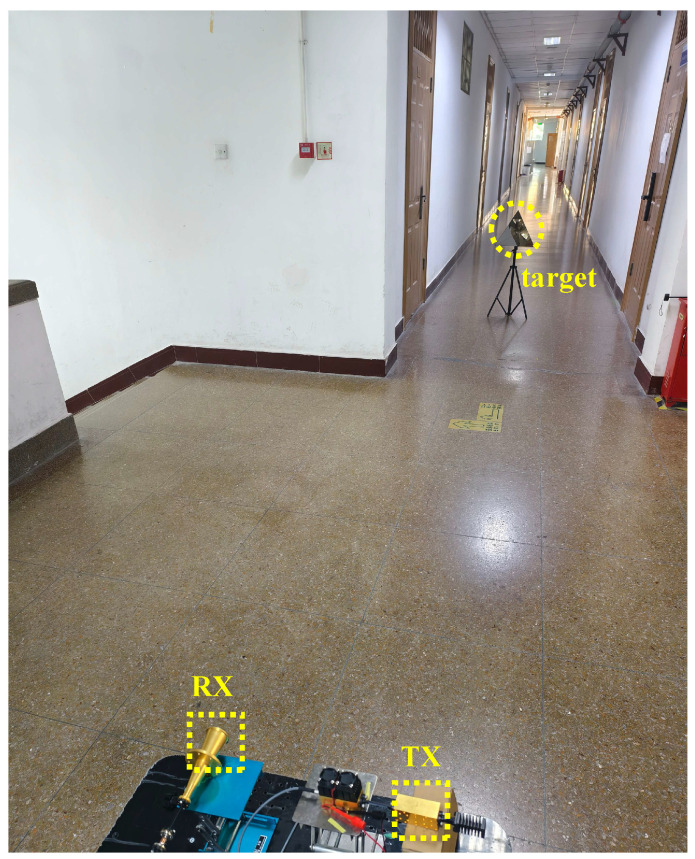
Radar system single-target test scenario.

**Figure 12:**
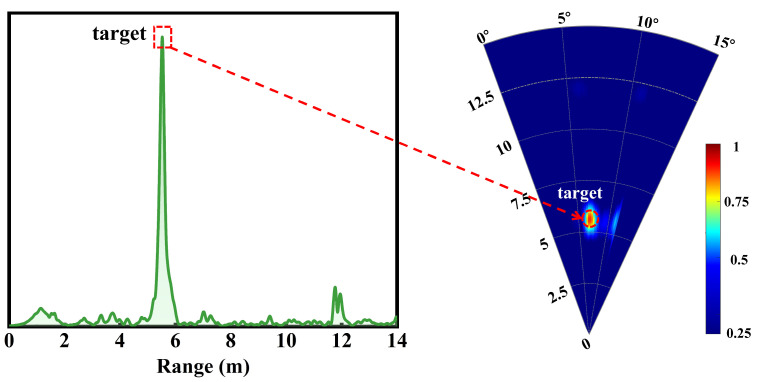
Result of the single-target radar test scenario.

We implemented a series of test scenarios to evaluate the feasibility of a through-wall radar system incorporating the designed antenna. The detection experiments used a linear frequency-modulated transmit signal that swept linearly from 93.6 GHz to 96.0 GHz with a chirp period of 1 ms, as shown in Figure 10. The resulting center frequency is 94.8 GHz, and the theoretical range resolution is ΔR = c/(2B) ≈ 6.25 cm. Received signals underwent downconversion, digitization, and processing using standard dechirping and Fast Fourier Transform (FFT) techniques to obtain beat-frequency spectra from which target ranges were estimated. Initially, a trihedral corner reflector served as a single calibration target and was positioned in an open corridor. The trihedral reflector used in the experiments has a radar cross section (RCS) of approximately 14 dBsm. The radar was fixed to the cart’s upper platform, with the antenna and reflector co-aligned at a height of 0.62 m above the floor. With the cart stationary, we defined the radar as the origin and the detection range as the straight-line distance to the target. Figure 11 and Figure 12 present the experimental configuration and detection results, respectively. The left panel of Figure 12 displays the range-amplitude map corresponding to the right panel; both distinctly indicate the presence of a single target, confirming the transceiver’s operational integrity. The radar indicated a range of 5.62 m, whereas a laser rangefinder measured 5.57 m, yielding a detection error of approximately 0.05 m.

**Figure 13:**
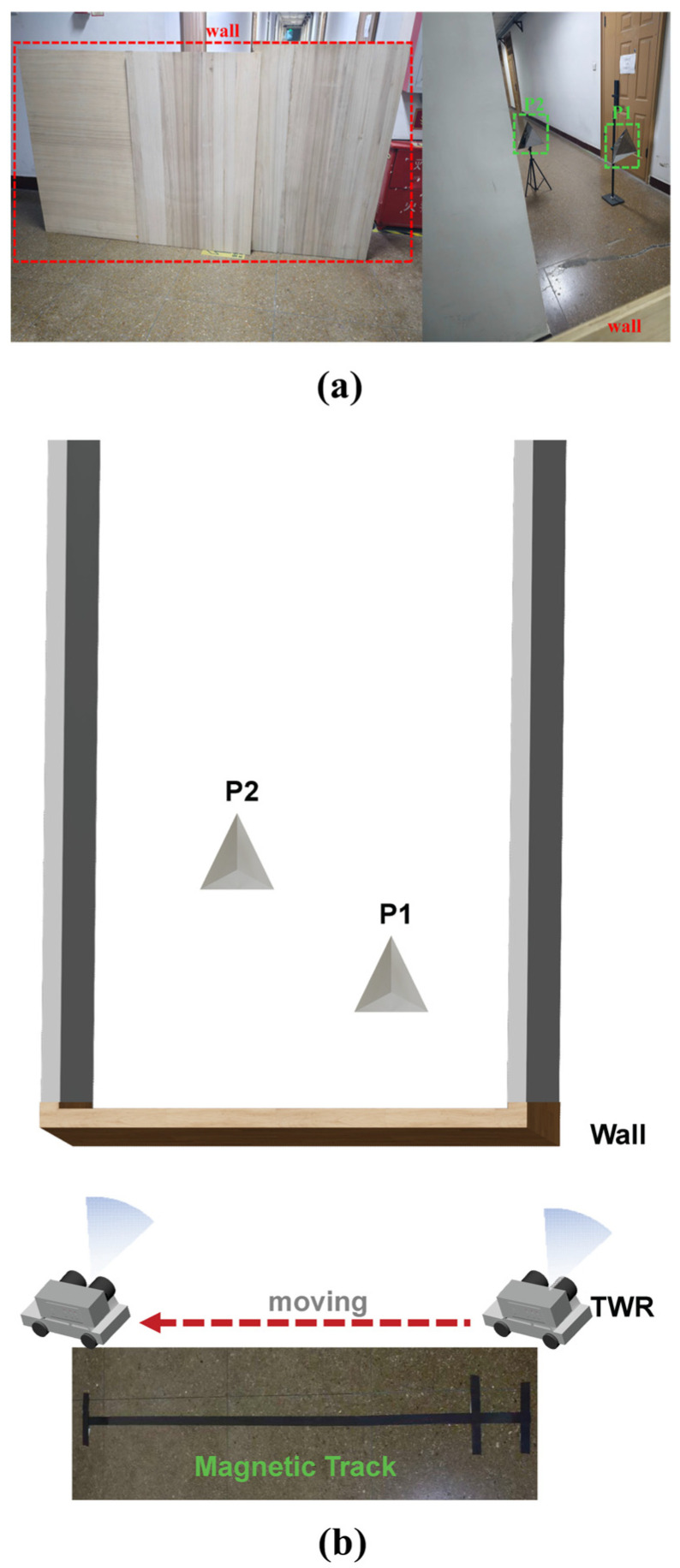
Schematic diagram of the multi-target through-wall radar test scenario. (**a**) Photograph of the test setup. (**b**) Corresponding schematic diagram.

After verifying the system’s integrity, we constructed a wall-penetrating test scenario, as illustrated in Figure 13. In this experimental setup, a wooden wall measuring 20 mm in thickness, 2.4 m in length, and 1.4 m in height served as an obstruction. The dielectric constant ε of the wooden wall is about 1.78, and the tangent loss tan δ is approximately 0.05. Two trihedral corner reflectors were positioned behind the wall to serve as targets, designated as P1 and P2. In this scenario, in addition to conducting stationary wall-penetrating radar detection, we performed experiments utilizing a vehicle-mounted system for wall penetration. The radar was securely fixed on the top platform of a small vehicle, and its movement path was controlled by a magnetic guideway laid on the ground. The vehicle moved parallel to the wall at a slow and constant speed of 13 cm/s, covering a total distance of 2 m.

**Figure 14:**
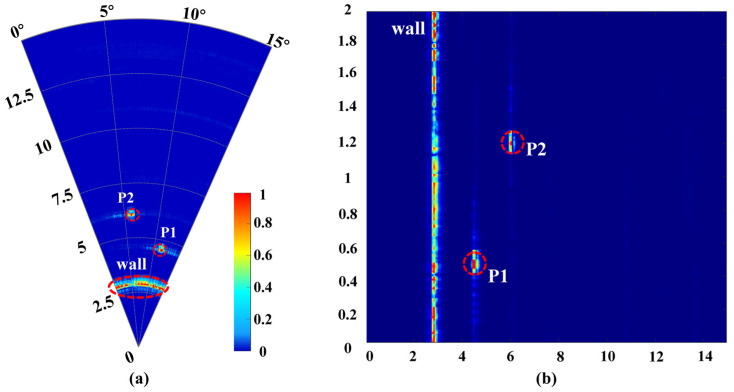
Result of the multi-target through-wall radar test scenario. (**a**) Acquired with the radar stationary. (**b**) Acquired with the radar mounted on a vehicle moving parallel to the wall.

Figure 14 presents the results of detection for both the stationary radar and the vehicle in motion. The linear distance from the vehicle to the targets corresponds to the lateral axis in Figure 14b (ranging from 0 to 14 m), while the direction perpendicular to the wall is designated as the initial angle in Figure 14a. The distance traveled by the vehicle corresponds to a vertical distance of 0 to 2 m. The results indicate that whether stationary or in motion, the TWR clearly depicted the continuously scanned wall and the two measurement targets located at different positions behind the wall. The test results showed that the wall was situated 2.95 m from the radar, which corresponds to an actual measured distance of 2.93 m, resulting in an error of 0.02 m. The distances for P1 and P2 from the radar were 4.5 m and 5.98 m, with actual distances of 4.47 m and 5.94 m, yielding errors of 0.03 m and 0.04 m, respectively. The along-track distance (in the direction of the vehicle’s movement) test results were 0.53 m and 1.21 m, with the difference between the two targets measuring 0.68 m, while the actual distances were 0.51 m and 1.16 m, leading to a difference in distance of 0.65 m and approximate errors of 0.03 m. The vehicle-mounted TWR completed the entire data collection process in about 16 s, effectively covering the detection area along the entire 2 m movement path.

**Figure 15:**
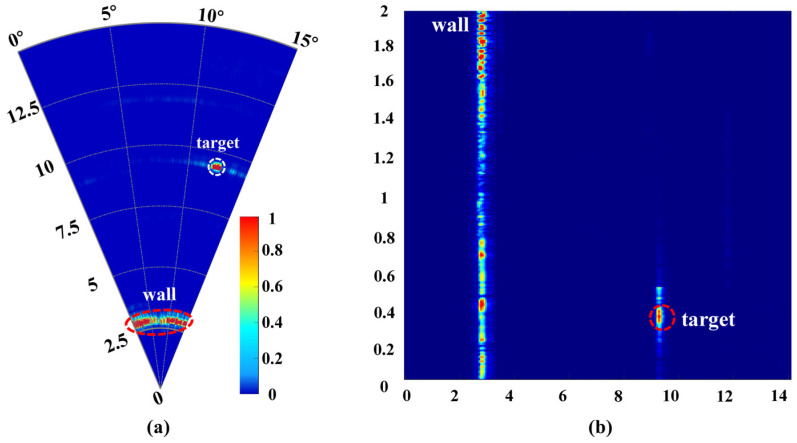
Result of the single-target through-wall radar test scenario. (**a**) Stationary. (**b**) Moving.

**Figure 16:**
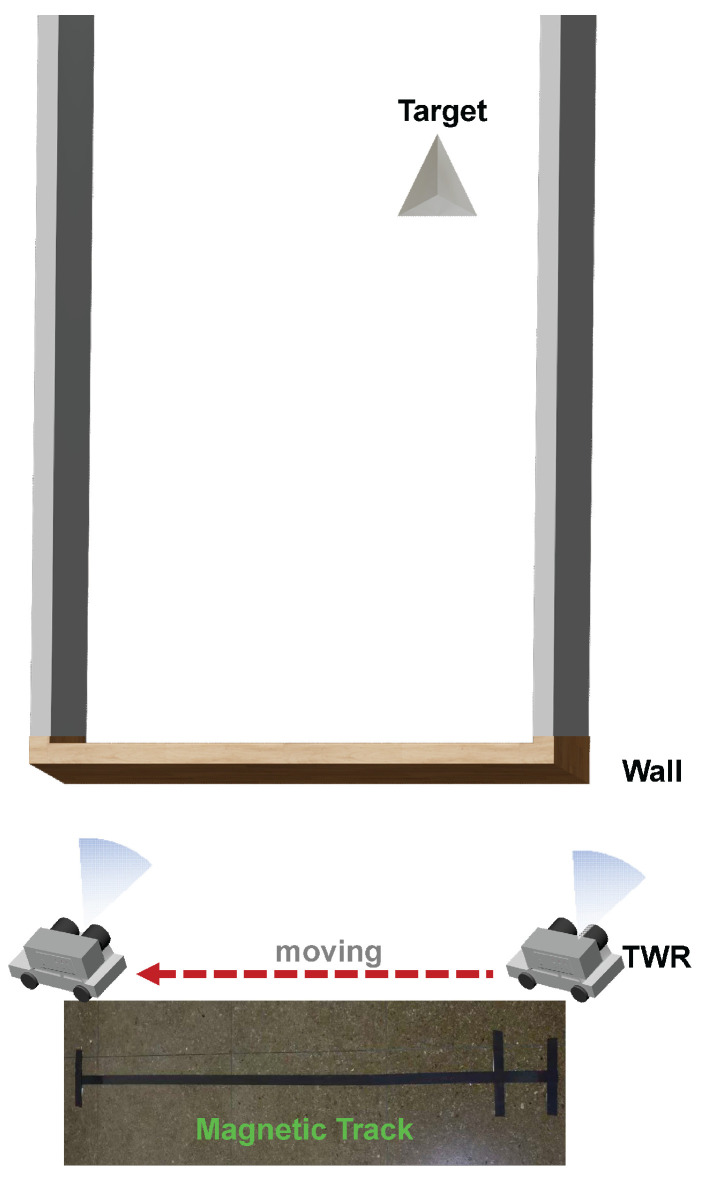
Schematic diagram of the single-target through-wall radar test scenario.

In this study, the radar’s maximum detection range is 10 m including the presence of the wall, as illustrated in Figure 15. To verify the 10 m detection distance, a trihedral corner reflector was placed approximately 10 m from the radar, as shown in Figure 16. The echo signals distinctly illustrated both the wall and the target to be measured, as depicted in Figure 15. The test results indicated that the target distance from the radar was 9.95 m, with a margin of error of 0.05 m, while the along-track distance measured 0.41 m, with an error of 0.02 m, and the actual distance was found to be 0.39 m. Through comprehensive testing across multiple scenarios, we thoroughly evaluated the performance and feasibility of the designed high-gain SSPP antenna and the W-band TWR system.

## 4. Discussion

Compared with conventional low-frequency TWR systems (sub-10 GHz), the proposed W-band solution addresses the need for higher range resolution and more compact radar front-ends by leveraging the wide instantaneous bandwidths available at millimeter-wave frequencies. Relative to other W-band frequency-scanning implementations based on waveguide or transverse slot arrays, the SSPP antenna design provides a fully metallic, low-dielectric-loss structure that is inherently more tolerant of high transmitted power and less prone to dielectric breakdown or surface discharge. Moreover, unlike multi-channel phased arrays, the SSPP frequency-scanning antenna requires neither complex phase-shifter networks nor mechanical rotation stages, substantially reducing system complexity, cost, and potential failure points—advantages that are particularly valuable for high-power, field-deployable TWR systems.

Integration of the antenna into a TWR and the successful detection of targets behind walls at ranges up to 10 m validates the practical utility of the approach. The CNC-machined, fully metallic prototype confirms manufacturability at W-band with acceptable tolerances. Vehicle-mounted and stationary trials further demonstrate that the antenna and radar architecture perform reliably under diverse deployment conditions, supporting applications such as search-and-rescue, structural inspection, and surveillance that require non-contact through-wall sensing.

Several limitations and challenges remain. First, the angular scanning range of frequency-scanned leaky-wave antennas is constrained by the operational bandwidth. To achieve broader angular coverage or finer control, additional bandwidth or multi-band strategies will be necessary. Second, the fully metallic SSPP architecture imposes stringent requirements on machining tolerances and surface finish at W-band; small geometric deviations can affect dispersion, radiation efficiency, and beam pointing. Third, system-level issues, such as thermal management of high-power W-band amplifiers, electromagnetic compatibility, and calibration in the presence of unknown wall dielectric properties, remain to be addressed.

In our future system improvement efforts, we will focus on the following aspects to broaden the applicability of the designed TWR. For the design of the radar’s transmitting and receiving antennas, we will consider employing bandwidth enhancement techniques to extend the scanning range of the controllable beam. Regarding radar system design, targeted optimization of the radio frequency front-end devices will be implemented to enhance reception sensitivity. Furthermore, signal processing techniques, such as clutter suppression, can significantly improve imaging performance, thereby increasing the adaptability and robustness for various wall types.

## 5. Conclusions

This article presents an innovative high-gain frequency-controlled beam scanning antenna. The antenna utilizes a sinusoidally modulated quasi-H-shaped SSPP structure based on the principles of SMRS theory to achieve radiation. By incorporating a flared section at the top, the antenna attains a peak gain of up to 20.9 dBi and exhibits a scanning rate of 4.05°/% within the frequency range of 92.8 GHz to 97.6 GHz. The fully metallic fabrication ensures superior power handling capability, while the prototype was fabricated using CNC machining. A W-band TWR system is developed based on this antenna and subjected to systematic validation. Prototype experiments were conducted across various detection scenarios, yielding results that confirmed the feasibility of employing this antenna for beam-scanning TWR applications. This design offers a straightforward and cost-effective transmitting antenna solution for high-frequency, high-power TWR applications.

## Figures and Tables

**Table 1:** SSPP Unit Heights within One Period (Unit: mm).

*h*_1_	*h*_2_	*h*_3_	*h*_4_	*h*_5_	*h*_6_	*h*_7_
0.41	0.48	0.58	0.63	0.58	0.48	0.41

**Table 2:** Performance Comparison of the Proposed Antenna and Related Works.

Ref.	Frequency (GHz)	Fabrication	Scanning Rate	Realized Gain (dBi)
[13]	84–109	CNC	0.77°/%	22
[14]	79–109	CNC	0.59°/%	17
[29]	77.5–85.5	PCB	1.47°/%	18
[22]	11.7–50	PCB	1.04°/%	10.9
**This work**	**92.8–97.6**	**CNC**	**4.05°/%**	**20.9**

## Data Availability

The original contributions presented in this study are included in the article. Further inquiries can be directed to the author.
